# Anatomical Targets Associated with Abrupt versus Gradual Washout of Subthalamic Deep Brain Stimulation Effects on Bradykinesia

**DOI:** 10.1371/journal.pone.0099663

**Published:** 2014-08-06

**Authors:** Scott E. Cooper, Klaus G. Driesslein, Angela M. Noecker, Cameron C. McIntyre, Andre M. Machado, Christopher R. Butson

**Affiliations:** 1 Cleveland Clinic, Center for Neurological Restoration, Cleveland, Ohio, United States of America; 2 Medical College of Wisconsin, Departments of Neurology & Neurosurgery, Biotechnology and Bioengineering Center, Milwaukee, Wisconsin, United States of America; 3 Case Western Reserve University, Department of Biomedical Engineering, Cleveland, Ohio, United States of America; Hospital Nacional de Parapléjicos, Spain

## Abstract

The subthalamic nucleus (STN) is a common anatomical target for deep brain stimulation (DBS) for the treatment of Parkinson’s disease. However, the effects of stimulation may spread beyond the STN. Ongoing research aims to identify nearby anatomical structures where DBS-induced effects could be associated with therapeutic improvement or side effects. We previously found that DBS lead location determines the rate – abrupt vs. gradual – with which therapeutic effect washes out after stimulation is stopped. Those results suggested that electrical current spreads from the electrodes to two spatially distinct stimulation targets associated with different washout rates. In order to identify these targets we used computational models to predict the volumes of tissue activated during DBS in 14 Parkinson’s patients from that study. We then coregistered each patient with a stereotaxic atlas and generated a probabilistic stimulation atlas to obtain a 3-dimensional representation of regions where stimulation was associated with abrupt vs. gradual washout. We found that the therapeutic effect which washed out gradually was associated with stimulation of the zona incerta and fields of Forel, whereas abruptly-disappearing therapeutic effect was associated with stimulation of STN itself. This supports the idea that multiple DBS targets exist and that current spread from one electrode may activate more than one of them in a given patient, producing a combination of effects which vary according to electrode location and stimulation settings.

## Introduction

The subthalamic nucleus (STN) is a common anatomical target for deep brain stimulation (DBS) for the treatment of Parkinson’s disease [1], and is being assessed for treatment of other disorders [2]–[4]. Over the past decade studies on subthalamic deep brain stimulation have looked for anatomical targets that optimize clinical outcomes [5]–[11]. However, the problem of relating electrode location to clinical results poses several challenges. First, although the location of the stimulating electrode contact within the brain can be determined from post-operative imaging, clinical effects derive not from that single point but from a surrounding *volume* of tissue activated (VTA) by the spread of electrical current, and this volume must be estimated. Second, the electrode is surrounded by multiple anatomical structures, each of which may be associated with different stimulation effects. Thus, the actual effects observed will be a mixture, reflecting overlap between VTA and different anatomical structures. Since many structures cannot be imaged *in vivo*, individual patient data must be coregistered with a detailed neuroanatomical atlas to identify these possible structures. Third, any data-based attempt at relating VTAs to clinical effects must employ some form of averaging whereby VTAs of multiple patients are combined to estimate a probability distribution function for clinical effects conditional on stimulation location.

We previously described two spatially distinct and therapeutically effective stimulation sites in the vicinity of the STN, distinguishable by the rate at which therapeutic effects on bradykinesia “wash out” after stimulation is turned off [12]. There was a statistically significant relationship between the location of active electrode contacts and the proportion of fast vs. slow washout. We found that when electrodes were located laterally most of the DBS effect disappeared abruptly, after which a small amount of residual effect washed out in a slow, gradual fashion. When electrodes were located medially, very little of the effect disappeared abruptly and most of it washed out slowly. This suggested that electrical current was spreading from the electrodes to two spatially distinct stimulation targets, with one or the other target activated preferentially according to electrode position. However, because of the difficulties enumerated above, we could not determine what neuroanatomical structures were actually responsible for the fast- and slow-washout effects. To address this, in the present paper, we use previously published approaches to identify these potentially different targets. We begin by estimating VTAs using patient-specific computational models [13]. Next, we coregister the VTAs with a detailed anatomical atlas [14]. Finally we combine VTAs from multiple patients to generate a probabilistic stimulation atlas (PSA) of bradykinesia washout effects [16], [17]. This approach illustrates how stimulation targets can be elucidated by combining outcomes from multiple patients.

## Methods

Design and conduct of the study was approved by, and written informed consent obtained per the Cleveland Clinic Institutional Review Board.

### Patients

14 Parkinson’s patients in [12] had sufficient perioperative clinical data available to reconstruct electrode locations for VTA computations; four subjects were excluded due to: 1) operated at another institution 2) “frameless” stereotaxic system used (incompatible with Cicerone software 3) incomplete surgical records and 4) incomplete radiological records. All had a diagnosis of PD by a movement disorders neurologist, clear levodopa response, and were non-demented. All had a minimum of 5 years disease duration and were at least 5 months post-implantation on the tested (dominant) side (range: 5–74 months). All had completed the initial postoperative period of DBS adjustments, and the median time since last adjustment was 14 months. Details of patient characteristics and stimulator settings appear in Table 1. Average values were (mean ± standard deviation): Age 61.8±5.9 years; Disease duration 14.5±5.2 years; Unified Parkinson’s Disease Rating Scale (UPDRS) OFF med/OFF stim motor score 30.9±11.3; preoperative total daily levodopa equivalent 1096±553.9 mg; percent reduction in daily levodopa equivalent from pre- to post-operatively (at time of testing) 46±34%; time since surgery 26.5±26.3 months.

**Table 1:** Patient characteristics.

Patient	Dominanthand	Age (years)	Disease duration (months)	UPDRSmotorOFF	Pre-operativelevodopa equivalent	% Levodopa reduction	Time since surgery(months)
A	right	62	27	28	1954	−47%	28
B	right	65	13	*	2148	−41%	74
C	right	58	19	50	1275	−57%	5
D	right	53	18	16	935	−6%	75
E	right	63	16	29	1250	−100%	70
F	right	72	9	28	0	0%	26
G	left	52	7	30	1239	−76%	11
H	right	69	12	13	1150	−58%	8
I	right	54	15	39	1193	−48%	16
J	right	64	12	43	1439	−48%	8
K	right	64	11	33	650	−54%	9
L	right	61	13	21	706	−60%	7
M	right	67	10	25	542	+29%	9
N	right	62	19	47	863	−77%	25

Dominant hand: right or left (all patients were tested with the dominant hand and contralateral STN stimulator). Age (years), at time of testing. Disease duration: time (years) from onset of Parkinson’s symptoms to time of testing. UPDRS motor OFF: UPDRS motor section total score in the off-medication, DBS-naive state. Preoperative levodopa equivalent: total daily levodopa equivalent (mg) preoperatively. % Levodopa Reduction: Percent change in daily levodopa equivalent from preoperatively to time of testing. Time since surgery: time of testing in months since electrode implantation. Missing data indicated by “*”.

### Experimental Procedure

Experimental procedure was described in [12]; we summarize here briefly as follows: Bradykinesia measurements were made at 2-minute intervals, and each measurement lasted 20 seconds. During each measurement, patients rapidly tapped the tips of the thumb and index finger together (UPDRS item 3.4 [17]). An angular velocity transducer (model G-1, NeuroKinetics, Edmonton, Alberta, Canada) was taped to the proximal index finger. As a measure of bradykinesia, we used total power in the angular velocity signal in the 1–10 Hz band (lower power = greater bradykinesia).

Patients came to the lab in the OFF-medication state, medications withheld for an average of 12 hours (range 10–16 hours). At the start of each experiment, DBS was ON at clinically optimized settings established prior to and independently of this study. Details of stimulator settings are provided in Table 2. Average values were (mean ± standard deviation): frequency 147.5±24.9 Hz; Pulse width 75±15.6 µsec, Amplitude 3.2±0.51 volts. 6 patients were stimulated in monopolar mode, 7 patients bipolar, and one had two negative and one positive contact.

**Table 2:** Stimulation Characteristics.

Patient	Electrode	Contacts	Frequency	Pulse width	Voltage
A	3389	1−,2−,3+	130	90	4.0
B	3387	1−,3+	135	90	3.5
C	3389	1−,C+	130	60	3.1
D	3387	1−,3+	185	90	3.6
E	3387	1−,C+	145	90	2.9
F	3387	2−,C+	185	90	2.8
G	3389	1−,3+	130	60	3.6
H	3389	2−,C+	130	60	2.8
I	3389	1−,C+	130	60	2.0
J	3389	1−,C+	130	60	2.8
K	3389	1−,3+	185	90	3.3
L	3389	2−,3+	135	60	3.6
M	3389	3−,2+	130	60	2.9
N	3389	1−,3+	185	90	3.5

Electrode Model: type of electrode implanted (Medtronic model 3387 or 3389). Contacts: electrode contacts stimulated (0–3, and “C” for “Case”). Frequency: stimulation frequency, in Hz. Pulse width: stimulation pulse width setting, in microseconds. Voltage: stimulation amplitude, in volts. These values represent each patient’s clinically optimized DBS settings which were chosen prior to, and independently of this study.

For twenty minutes, bradykinesia was measured every two minutes. Then the stimulator contralateral to the dominant hand was turned off, and measurements continued every two minutes for a further 50 minutes. At the conclusion of this time, the stimulator was turned back on, and measurements were again made every two minutes for a further 20 minutes.

The following procedure was used for turning off/on the stimulator: Patients were told beforehand that their stimulator settings would be changed at some point during the experiment, but the exact nature and timing of the change was obscured from them by having the experimenter press ineffective buttons on the programmer device at random while the patient performed a distractor task (visual choice reaction time task) for 4 minutes. Half-way through this procedure, the real, effective button was pressed which turned the stimulator off/on.

**Figure 1:**
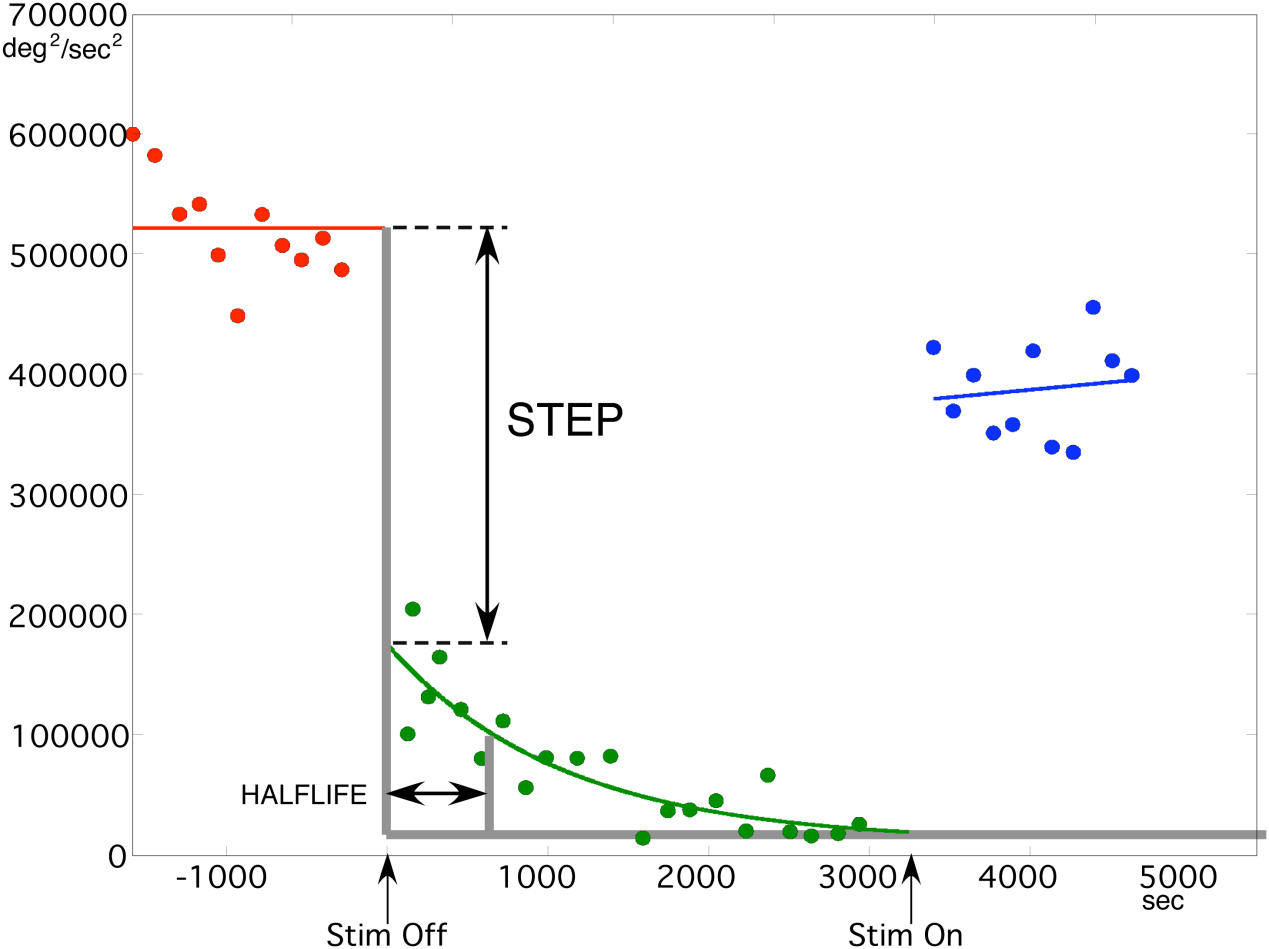
Data from one patient, showing the decaying exponential fit to bradykinesia measurements after DBS was turned off, and the abrupt change in bradykinesia quantified by %STEP, followed by further slow washout of residual therapeutic effect. In this patient, about two-thirds of the total bradykinesia returned abruptly, while the remaining one-third washed out slowly, giving a value for %STEP of 67%.

Curves were then fit to the bradykinesia versus time data using Nelder-Mead iterative minimization of summed, squared error [18]. An example is shown in Figure 1. The abrupt loss of stimulation effect appears as a discontinuity between the curve during the initial stimulation-on baseline interval, vs. during the stimulation-off interval. The further, gradual decrease from that time to the eventual plateau represents the slow washout of residual therapeutic effect. Of the total change in bradykinesia, from baseline to plateau, the percent which occurred abruptly was measured by the “%STEP” parameter.

The curve fit to the stimulation-off interval was a first-order exponential function of time. We found that the curve fit to the stimulation-on interval could be either first-order exponential, first-order linear, or zeroth-order linear function of time without affecting the relationship between electrode location and the %STEP parameter. In the present paper, for the baseline interval, we use the zeroth-order linear function (i.e. a simple mean of baseline values).

### Patient-Specific VTA Models

**Figure 2:**
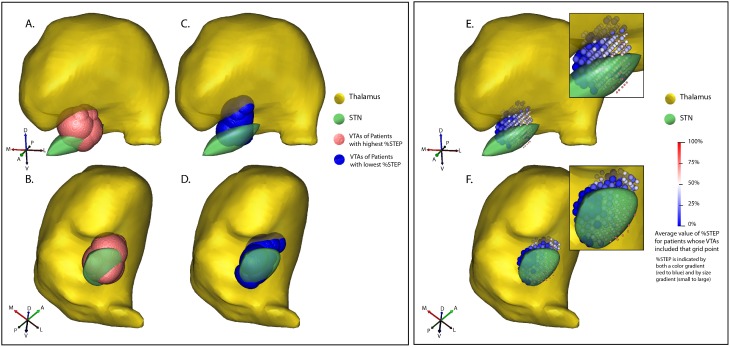
Estimated volumes of tissue activated (VTA) for the 7 patients with the most rapidly-decaying DBS effect, i.e. highest %STEP (mean ± sd 58±21% A, B; viewed from two different angles) and the 7 with the most slowly-decaying DBS effect, i.e. lowest %STEP (mean ± sd −8±28%; C, D, from the same two angles). E–F Spatial average of data from all 14 patients. Size and color of each grid point represent the average value of %STEP for all patients whose VTA included that point.

We generated patient-specific computational models of DBS with Cicerone v1.2 software, a freely available academic DBS research tool [19]. In the first step, preoperative MRI, CT (with stereotaxic frame fiducials), intraoperative microelectrode recordings, postoperative CT (with electrode), and a volumetric atlas containing thalamus and basal ganglia are co-registered, to give an estimate of electrode location in relation to those structures. Image registration in Cicerone is performed using a previously published mutual-information algorithm [20] which is used extensively in many commercial and open-source image imaging programs. In this case, each patient’s pre-operative MRI and post-operative CT were coregistered, along with registration of the patient MRI and the atlas MRI. Next, based on a finite-element model of the electrically conductive tissue surrounding the electrode, an estimate is computed of the electric field in spatial relation to the neuroanatomical structures surrounding the electrode, using the stimulation parameters (electrode contacts, voltage, pulse width, and frequency) for each patient. Finally, based on a multicompartment-model of excitable neuronal elements, with detailed simulation of ion channel dynamics using Hodgkin-Huxley formalism, a volume of tissue activated (VTA) is computed [21], estimating the region within which stimulation produces action potentials (Figure 2).

### Atlas Registration

**Figure 3:**
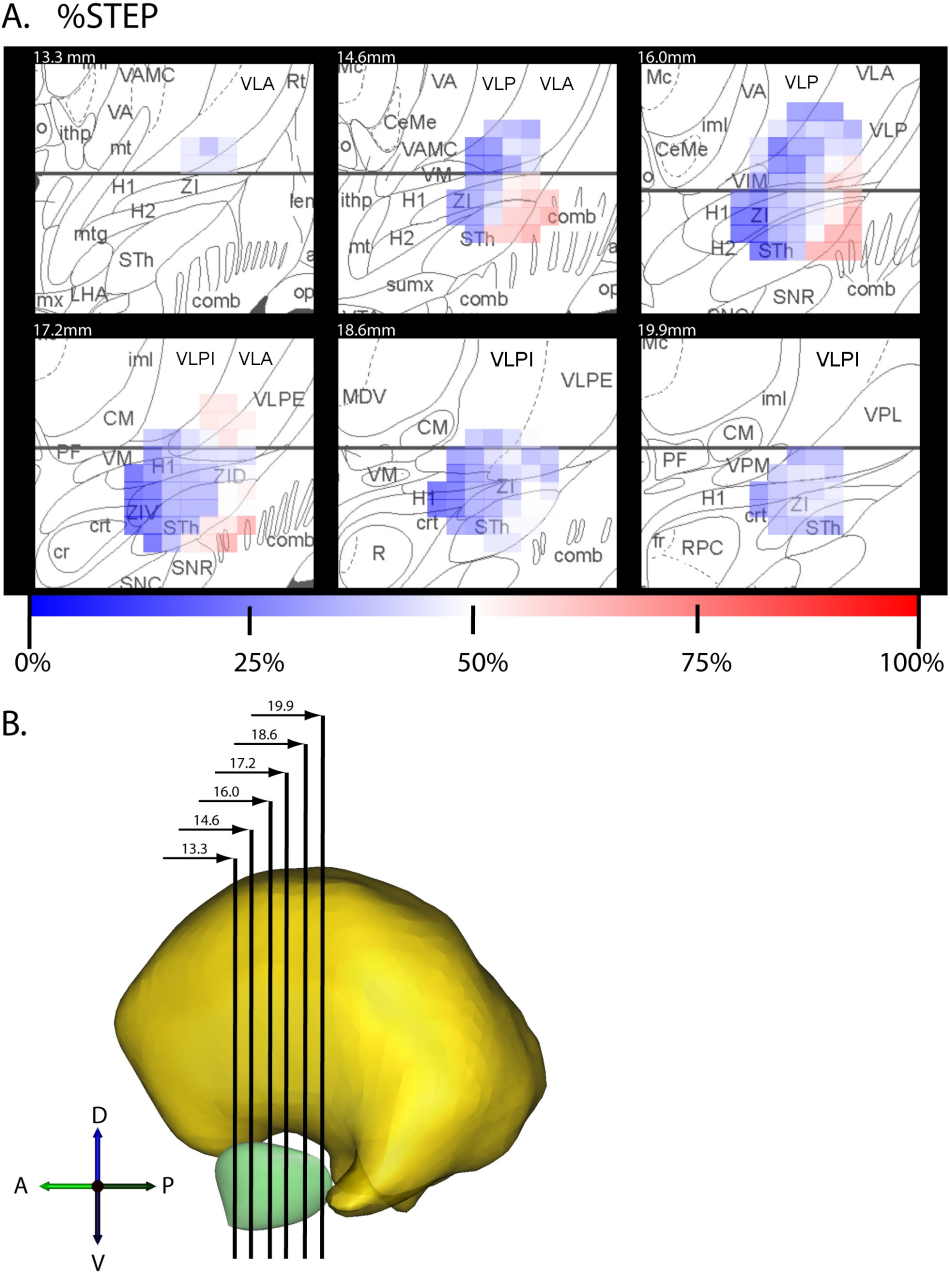
A) Slices corresponding to Mai et al [15] atlas sections were taken through the 3-dimensional field representing the average value of %STEP over the 14 patients. B) Average value of %STEP is shown in false-color, in relation to the structures delineated in the atlas.

The brain atlas used in Cicerone provides surface representations of the thalamus and STN, but lacks representations of other smaller anatomical regions that were of interest for the present study. To address this limitation we coregistered each patient with a detailed anatomical atlas of Mai et al [14] that delineated zona incerta (ZI), fields of Forel (FF) and other nearby structures. We extracted the following structures from each applicable page of the Mai et al atlas [14]: thalamus, H1, H2, STN, ZI, anterior commissure, posterior commissure. First, distinct grayscale values were assigned to the nuclei in each slice using Photoshop (Adobe Systems Inc, USA). Then outlines of each nucleus in each slice were imported into Mimics (Materialise HQ, Leuven, Belgium) at relative positions determined from the slice thicknesses specified in the atlas (e.g. see figure 3B). We then used the outlines of each nucleus to create a closed polygonal surface, and loaded the surfaces and the original atlas slices into SCIRun (SCI Institute, University of Utah), which also contained the original nuclei surfaces from Cicerone. To register the two atlases, we first confirmed the alignment of both groups of nuclei (x-direction = medial-lateral, y-direction = anterior-posterior, z-direction = dorsal-ventral), and scaled the Mai-Paxinos atlas slightly in the anterior-posterior direction to provide precise alignment of the anterior and posterior commissures. Next we used a previously-published [21] minimum least squares method in Matlab (Mathworks Inc, Natick, MA) to align the STN and thalamus from the Mai et al and Cicerone atlases (these nuclei were chosen because they were common to both atlases). This method allows translation and rotation (shear and scaling were not permitted) of one set of nuclei relative to another set of nuclei in order to minimize the total “error distance” between the pairs of thalamus and STN surfaces. The distance was measured from the normal vector from each surface point in the Cicerone atlas to the location where it intersected the analogous surface in the Mai-Paxinos atlas. Once this transformation matrix was determined, it was applied to all surfaces and slices from the Mai-Paxinos atlas to position them in the Cicerone atlas space. These registration steps were necessary for integration of all patients’ VTAs into a common atlas space that included ZI, FF, and STN boundaries.

### Probabilistic Stimulation Atlas (PSA) [15], [16]

Finally, we combined all 14 patients’ bradykinesia wash-out data into the common anatomical space. This was done by creating a 3D lattice that was large enough to contain the VTAs from all patients. The lattice was constructed from 1 mm^3^ voxels with overall dimensions of 30 mm×30 mm×30 mm. Each VTA was used to mask the voxels contained within it, and each of those voxels was assigned values for %STEP. From these individual scores, average values were computed for voxels that were contained within two or more VTA. This analysis was performed using Matlab (Mathworks Inc, Natick, MA).

## Results

Figure 1, shows how we quantified the amount of abrupt decrease in stimulation effect, relative to the subsequent slow-decay with the %STEP parameter. Large values of %STEP (predominantly the fast-decaying process) occurred with laterally located electrodes and small values (predominantly the long-lasting process) with medial ones.

Figure 2 shows VTAs for the 7 patients with highest (A,B, seen from two different angles) vs. 7 lowest (C,D, from the same two angles) values of %STEP. VTAs for patients with predominantly slow-decaying effect are clustered medial to the STN, while VTAs for patients with predominantly fast-decaying effect are centered on the STN proper. Some VTAs extend dorsally into the thalamus, apparently following the path of pallidothalamic fibers located medial to STN to their thalamic terminations. On the other hand, this may reflect bipolar stimulation (see Table 2). When a patient is stimulated with a ventral cathode and a dorsal anode, the VTA will include activation by *both* contacts, and cannot determine whether the more dorsal contact contributes to that patient’s %STEP value, *vs.* %STEP being wholly determined by the ventral contact, with no contribution from more dorsal activation in thalamus.

Whereas the most slow-decaying VTAs were confined to a medial zone, the fastest-decaying VTAs included both lateral and medial regions. Consistent with this observation, none of the patients in our sample exhibited a pure fast-decaying effect whereas a pure slow-decaying effect was seen in some cases. That is, some patients in our sample had a %STEP value of 0% (zero percent fast, 100% slow), whereas even the most fast-decaying patients had a %STEP value of 85% (85% fast, 15% slow). This asymmetry is likely explained by the lack of far-laterally located electrodes in our sample. Far-laterally located electrodes activate corticospinal and corticobulbar fibers, causing side effects, and such patients were rare in our study, because we enrolled only patients with good clinical response to the surgery. Therefore, in our sample, whenever a VTA included lateral STN, the electrode tended to be located in central or medial STN, with activation spreading to lateral STN. Since, with increasing stimulation amplitude, activation spreads both medially and laterally, such VTAs tend to encompass a large mediolateral range. In contrast, medial electrode placement was less constrained by side effects, and so our sample includes patients with relatively selective medial stimulation and pure slow-decaying effect.

In order to provide a more detailed identification of stimulation targets we generated a PSA that averaged data across VTAs from all patients (Figure 2 E,F). The resulting scalar field is represented as a regular grid, with size and color of each grid point representing the average value of %STEP for all patients whose VTA included that point. The lowest values of %STEP (the most slow-decaying DBS effect) are located medial to the STN (large blue grid points), while higher values (more fast-decaying effect) occur in STN proper (small red grid points).

Figure 3 shows the scalar field in the space of the detailed anatomical atlas [14]. We found that the slow-decaying region (lower values of %STEP: blue) is centered in the vicinity of posterior zona incerta and fields of Forel, extending dorsally into ventrolateral thalamic nucleus (VLA). In contrast, larger values of %STEP (red) occur in STN proper.

**Figure 4:**
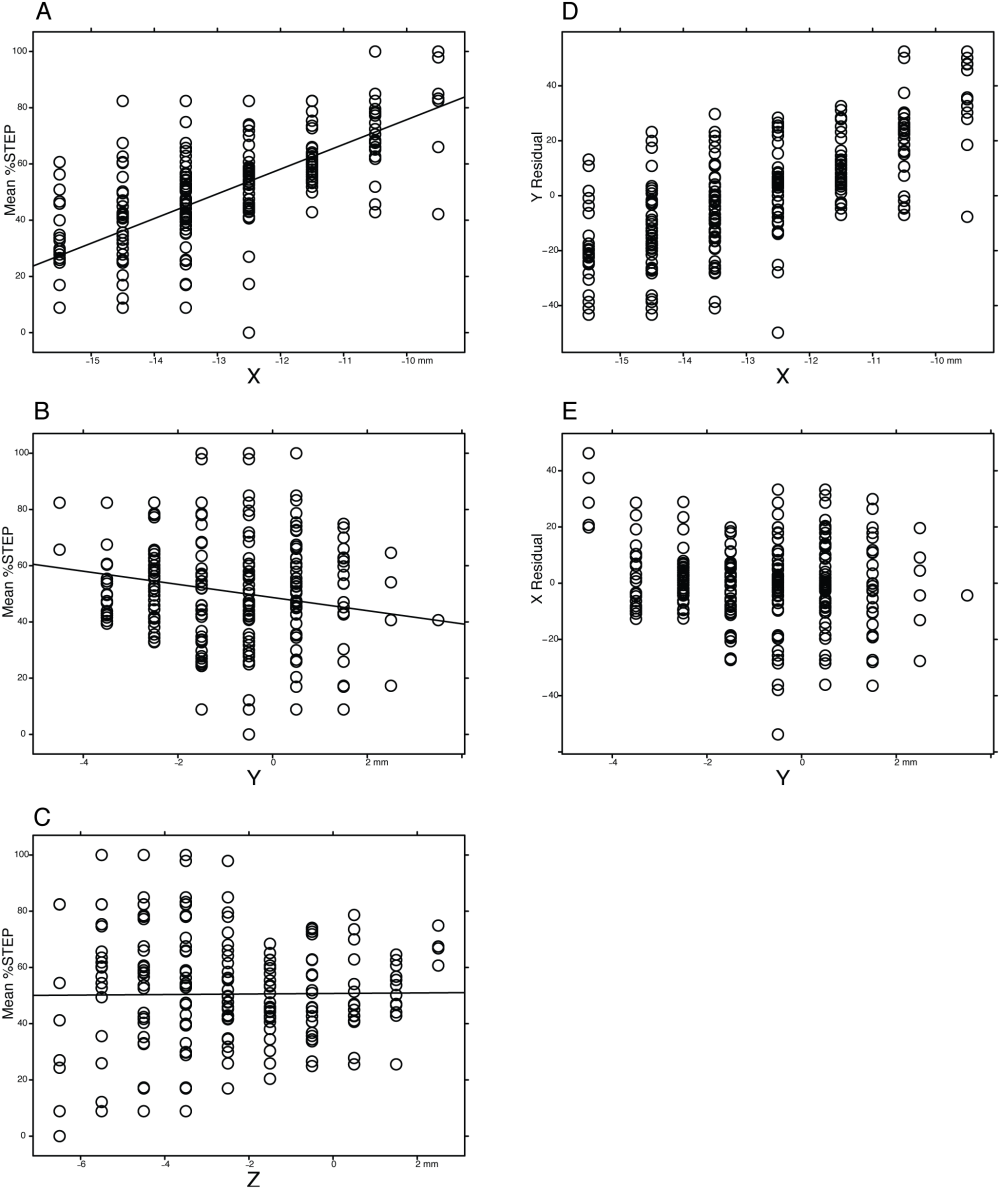
Mean %STEP vs stereotaxic coordinate in stereotaxic space. Each point is a grid point as shown in Fig. 2 E, F. Figure 4 A, B, C: simple regressions on X (mediolateral), Y (anteroposterior), & Z (dorsoventral) coordinate. Figure 4 D, E: residual variances. Fig. 4 D shows the effect of X coordinate when Y is partialed out, and Fig. 4 E shows the effect of Y when X is partialed out. Note that %STEP is related to both X and Y, however, the effect of X is independent of Y, whereas the converse is not true.

Could the %STEP gradient (blue to red gradient in Figure 3) have arisen under the null hypothesis that %STEP is unrelated to which neuroanatomical structures are stimulated? To answer that question, we performed the regression analysis in Figure 4. In this analysis, each data point is a single grid point in Figure 2 E, F. As shown in Figure 4, there was a strong dependence on mediolateral location of stimulation (Fig. 4 A, Bonferroni-corrected p<0.001), and on anteroposterior location of stimulation (Fig. 4B, Bonferroni-corrected p<0.01), and no dependence on the dorsoventral axis (Fig. 4C, p>0.5). Thus, our observations have very low likelihood under the null hypothesis. A closer examination of the data shows that the effect is driven by mediolateral (X) location. This may be seen by direct comparison of Fig. 4A with Fig 4B, as well as by the following procedure, shown in Fig. 4 D, E: When variance predicted by Y is partialed out of %STEP, and the residual regressed on X the relationship remains clear (Fig. 4D), whereas, with the inverse procedure (variance predicted by X partialed out, and residual regressed on Y) the relationship is lost (Fig. 4E).

This analysis significantly extends our earlier result in which %STEP was shown to be related to electrode contact locations [12]. In that analysis, contacts stimulated at low or high voltage, with positive or negative polarity were treated the same: no allowance was made for the difference between anodal vs. cathodal stimulation or the differential spread of current with higher vs. lower amplitude stimulation. In the present analysis, these factors are taken into account by the VTA calculation, and the %STEP gradient is again seen.

## Discussion

### Clinical Implications

A growing number of publications suggest that DBS clinical outcomes reflect subtle differences of targeting in and around the STN. The classical target is the dorsolateral-anterior STN, where neuronal activity is related to passive and active limb movement [22], and related anatomically (in animal models) to motor cortex [23]. However, different locations of stimulation may give difference in side effects [10] differential effectiveness for different symptoms [22], [24], [15], or difference in both respects [25]. The present study distinguishes between medial (Zona incerta/Fields of Forel, ZI/FF) and lateral (STN proper) targets based on rates of washout, but does not attempt to assess their relative clinical merits: we do not conclude that one target is better than the other; neither do we conclude that they are equally good. Other studies address this: Clinical [5]–[8] and postmortem [26], [27] studies suggest that stimulation just outside the STN proper in ZI/FF may give clinical outcomes as good as or better than stimulation within the nucleus. One potentially useful conclusion suggested by our results is that intermittent stimulation may be more effective with the medial target. Existing DBS implantable pulse generators (IPGs) can be programmed to deliver stimulation alternating with “stimulation-off” periods (and the newest generation of IPGs approved for DBS in the United States allows this without safety concerns related to charge imbalance). If the “off” intervals are short, relative to the washout halflife, this could give similar clinical benefit to continuous stimulation, but with lower power consumption, prolonging battery life. Our results suggest a more medial target, if such a strategy is attempted.

What does seem clear, though, is that DBS targeting has progressed beyond the stage of aiming for a visually conspicuous histological or atlas feature, and is now concerned with finding the best target within an anatomically complex region. The strength of this view derives from the variety of different techniques supporting it: in-vivo reconstruction of contact locations in comparatively large series, post-mortem histological location of contact locations in individual cases, and computation of volumes of tissue activated (VTAs).

### Potential DBS mechanisms

Can we associate particular physiological processes with our fast- and slow- decaying processes? The current state of knowledge leaves wide scope for speculation: for example a recent review [28] lists 11 hypothetical mechanisms whereby DBS may alleviate symptoms, arranged according to the time scale on which their effects would occur. The bulk of research on Parkinson’s STN DBS mechanisms has focused on stimulation’s ability to directly depolarize neurons, overriding the spontaneous, pathological pattern of activity [29]. Such a mechanism would be expected to have very rapid onset/offset, on the order of milliseconds, hence fits well with our “fast” mechanism. According to the classical view, this mechanism operates orthodromically, exciting action potentials via the glutamatergic subthalamopallidal synapse [30], while, according to a novel view supported by optogenetics [31] see also [32] it operates antidromically exciting cortex via the hyperdirect pathway [23]. In either case, the effect would be evoked from STN proper, which is where we find our “fast” effect.

Less research exists on possible DBS mechanisms whose washout might fit our “slow” process; however, Lee [33] has proposed extracellular neurotransmitter accumulation as a DBS mechanism, and this appears [34] to exhibit washout on the correct time scale for our slow process. Our slow process originates from a zone in the vicinity of posteroventral ZI, H1, and H2. Forel’s fields H1 and H2 are fiber tracts, carrying pallidal efferents; in contrast, ZI is a nucleus with wide projections, including to thalamus, substantia nigra, and spinal cord [35]. Since direct stimulation of GPi improves PD symptoms [36], Parent & Parent [37] have suggested that stimulation of pallidothalamic fibers in the fields of Forel may have a similar therapeutic effect; this may explain why the zone of long-lasting DBS effects extends into ventrolateral thalamus where pallidothalamic fibers terminate. Hypothetically, extracellular neurotransmitter accumulation and reuptake at pallidothalamic synapses, incerto-thalamic synapses, or in ZI proper might produce the slow-washout effect, but available data do not allow us to do more than speculate.

### Limitations

Our findings must be interpreted in the context of known limitations in our approach. First, no perfect or universally-accepted method exists for determination of electrode locations. We used Cicerone software [19] to define the electrode location relative to the individual patient anatomy. This particular technique has proven to be effective in numerous previous studies e.g. [10], [12], [37]–[40], nonetheless uncertainty is associated with each patient. These types of errors or uncertainty exist for all forms of DBS electrode location estimation in human brains [41], [42]. However, a new addition from this study was the integration of the Mai et al. atlas within the context of Cicerone. This allowed us to visualize additional anatomical structures, but required an additional registration step which introduced an additional degree of error into our analysis. Direct quantification of this error is impossible, because no ground truth point exists for the dataset; however, we did attempt to minimize the error by using a least squares optimization algorithm to fit the Mai et al. atlas to each patient’s Cicerone model.[10], [12], [38]–[40].

Second, since VTAs cannot be imaged directly, estimation of VTAs requires computational models, which introduce uncertainty. The methods we used to generate VTAs have been tested in prior studies [21]. Finally, we have attempted to locate our estimated VTAs in relation to anatomical structures which are quite small, and whose spatial relationship to each other is complex. We chose the atlas of Mai et al because it provides a high level of detail for the fields of Forel and zona incerta, and coregistered VTAs to that atlas using common landmarks (thalamus and STN). Nonetheless, this procedure must be regarded as approximate at best. The complex interdigitation of ZI, H1, and H2 in the slow-decaying target zone makes it unlikely that VTA studies alone can tell us which of them is responsible for therapeutic DBS effects evoked from that region, although, in future fiber tract activation modeling [40] may offer a way to disentangle them.
